# Knowledge, attitude and practices towards visceral leishmaniasis among HIV patients: A cross-sectional study from Bihar, India

**DOI:** 10.1371/journal.pone.0256239

**Published:** 2021-08-17

**Authors:** Devipriya J. S., Ashok Kumar Gupta, Rajendra Babu Veeri, Pavan Garapati, Rishikesh Kumar, Sameer Dhingra, Krishna Murti, V. Ravichandiran, Krishna Pandey

**Affiliations:** 1 Department of Pharmacy Practice, National Institute of Pharmaceutical Education and Research (NIPER), Hajipur, India; 2 Department of Pharmacy, School of Medical and Allied Sciences, Galgotias University, Greater Noida, Uttar Pradesh, India; 3 Department of Biostatistics, ICMR-Rajendra Memorial Research Institute of Medical Sciences (RMRIMS), Agamkuan, Patna, India; 4 Department of Clinical Medicine, ICMR-Rajendra Memorial Research Institute of Medical Sciences (RMRIMS), Agamkuan, Patna, India; Curtin University, AUSTRALIA

## Abstract

**Background:**

In the Indian state of Bihar, visceral leishmaniasis (VL) is a major public health issue that has been aggravated by the rising incidence of new Human immunodeficiency virus (HIV) infections. In endemic areas, the risk of VL infections in patients living with HIV (PLHIV) is higher. It is important to investigate the disease-related knowledge, attitude, and practices (KAP) of PLHIV in Bihar in order to monitor HIV/VL co-infection. Adequate knowledge, a positive attitude, and good practices for VL control are essential to stamp out the disease. This study investigated the KAP towards VL in HIV patients attending antiretroviral therapy (ART) clinic at ICMR-RMRIMS, Patna.

**Methods:**

A questionnaire based cross-sectional study was performed among 120 HIV patients aged ≥18 years, to evaluate their KAP regarding visceral leishmaniasis. For the KAP indicators, each correct answer received a score of 1, while unsure and incorrect responses received a score of 0. Descriptive statistics and logistic regression were used for the analysis. Data analysis was performed using Statistical Package for the Social Sciences (SPSS) version 27.

**Results:**

The study population had a male (68.30%) preponderance with a mean age of 37.03 years ± 9.80 years of standard deviation. The majority (93.30%) of the study participants had previously heard about VL. Only 32.10% of those who had heard about VL knew that the disease was transmitted by the sandfly. Most (80.40%) of the study respondents were ignorant of the sandfly breeding grounds. The vast majority (75.90%) had no idea how to recognize sandflies and were unaware of their biting time, leishmaniasis transmission season, and preventive practices. Although PLHIV are vulnerable to VL, only 27.70% of them agreed that VL is a fatal disease if untreated, and 42.90% believed they wear not at risk of developing the disease. Regarding the control methods of sandflies, 28.60% of participants did not use any methods to avoid sandfly bites. The multivariable analysis revealed that occupation and family history were the two independent predictor variables of the knowledge index. Age and gender were significantly associated with attitude towards VL. Participants working as laborers had significantly lesser odds (AOR: 0.248, 95% CI: 0.073–0.844) to follow good preventive practices. There were significantly higher odds of having good practice among participants aged 18–40 years (AOR: 6.866, 95% CI: 1.694–27.834) and those residing in urban areas (AOR: 4.159, 95% CI: 1.317–13.139) than their peers. Overall, 27.7% of respondents were knowledgeable, 41.1% had a positive mindset, and 33.9% had strong VL preventive habits, according to the study.

**Conclusion:**

The study determined a remarkable gap in the knowledge attitude and practices towards VL among PLHIV. This underscores the need of augmented health education initiatives for PLHIV in endemic areas for good VL awareness and preventive practices.

## Introduction

Visceral leishmaniasis (VL) is the most lethal form of leishmaniasis caused by a protozoan parasite *Leishmania donovani* complex. Transmitted by the bite of *Phlebotomus argentipes;* a female sandfly with anthroponotic infection in the Asian subcontinent [1]. Visceral leishmaniasis is characterised by frequent episodes of fever, hepatosplenomegaly, pancytopenia, weight loss, and anemia [2]. Every year, 700,000 to 1 million new cases of VL are reported worldwide, with more than 95% of them occurring in ten countries: Brazil, China, Ethiopia, India, Iraq, Kenya, Nepal, Somalia, South Sudan, and Sudan [3]. In South-East Asia, the three most affected countries are India, Bangladesh, and Nepal, with more than 50% global burden [4]. In India, VL remains a paramount public health concern with confirmed endemicity in 54 districts across four neighboring states, i.e., Bihar, Jharkhand, West Bengal, and Uttar Pradesh [5]. Bihar alone accounts for 40% of the worldwide burden of VL and 90% of India’s total registered cases [6]. Bihar has always been the epicenter of VL infections for over a century; at the same time, the incidence of new Human Immunodeficiency Virus (HIV) infections are also increasing [7]. The prevalence of HIV and VL co-infection in India varies between 2 to 5.6%, according to several published reports [6, 8].

HIV Patients residing in VL-endemic areas have a greater than expected chance of contracting the disease [9]. PLHIV have a 100 to 2320 times higher chance of developing active VL than HIV-negative people [10]. VL increases HIV replication and disease progression while HIV can reactivate leishmaniasis infection [10]. HIV-VL co-infection results in delayed diagnosis, frequent relapses, poor treatment outcomes, and premature deaths [11, 12]. The overlapping of HIV and VL results in the ruralization of HIV and urbanization of VL [13]. The Indian government has set the goal of eliminating VL as a public health crisis by 2020, under the control of the world health organization (WHO) [14]. Patients with HIV-VL co-infection can act as super-spreaders of VL, posing a significant threat to on-going VL elimination efforts [12].

HIV-VL co-infection has emerged as a serious health concern and it could seriously complicate the state’s attempts to eliminate VL. In this scenario, the adoption of better/good preventive strategies depends on their knowledge, attitudes, and behavior towards the disease. To control HIV-VL co-infection, it is essential to study the disease-related KAP of PLHIV in Bihar. Over the years, various studies have been conducted in the endemic population to evaluate the VL related KAP, revealing poor knowledge about symptoms, infectious nature, mode of transmission, and preventive measures of VL [15–18]. Despite the fact that PLHIV is more susceptible to VL, no research has been conducted to determine their KAP. The synergistic detrimental effect of both diseases demands special attention on PLHIV to prevent co-infection related comorbidities. Lack of awareness among HIV patients about VL may result in high transmission. Knowledge of VL is a prerequisite for developing preventive strategies.

We are unaware of any studies that evaluated the knowledge, attitude, and practice of VL in PLHIV in Bihar. The findings from this research can be used to build public initiatives in Bihar to minimize HIV-VL co-infection. Therefore, this study is designed to evaluate the knowledge, attitude, and practices regarding VL among people living with HIV/AIDS.

## Methods

### Study site and design

A hospital-based cross-sectional study was performed to analyze the KAP towards VL in PLHIV in Bihar. RMRIMS is a tropical disease research center cum hospital under ICMR, a hub of clinical research-related activities in VL in Bihar. RMRIMS is the largest referral center in the state, for VL treatment. It also provides Integrated Counselling and Testing Centres (ICTC) for HIV counseling and testing. Confirmed HIV patients of both genders aged > 18 years were included in this study. Patients with known/reported opportunistic infections, chronic diseases, or psychological disabilities were excluded from the study.

### Sample size

The sample size was calculated using Open Epi software Version 3.01. Since the prevalence of KAP in the study population was unknown; a pilot study was carried out to determine the feasibility and sample size of the study. Based on the findings of this pilot study, the proportion of patients with accurate disease knowledge, a positive attitude, and preventive practice was 4%, 4%, and 8%, respectively, with a confidence interval of 95%, and a margin of error of 5%, the required sample size was assessed to be 59, 59 and 112 respectively. However, to have a more representative sample, we recruited 120 people in the study. A convenience sampling method was used to recruit HIV patients who visited the ART clinic between January 2019 and January 2020.

### Data collection and data analysis

A well-structured, pretested questionnaire was used to collect data. All the questions were derived from published studies that have assessed KAP about VL in non-HIV populations [15–18]. The questionnaire consists of 4 sections that address the 1] socio-demographic information 2] knowledge and awareness of participants where VL was concerned 3] Attitude of participants towards VL 4] Preventive practices related to VL. A trained research assistant administered the questionnaires in Hindi. To avoid stigma and improve participation, all interviews were organized in a private room. The questionnaire was pretested in 10% of the subjects to ensure data reliability, and this data was omitted from the final study. The data was verified by the principal investigator for accuracy and completeness before use. The questionnaire was scored based on the methods used by Berhe et al. 2018 [17]. Each response was scored one for the correct answer while zero for the wrong/ “don’t know” response. knowledge about VL was graded on a scale of 0 to 8, with 0 being the lowest and 8 being the highest. Participants’ scoring from five to eight was considered as "good knowledge" while zero to four score indicated "poor knowledge". Similarly, attitude/perception about VL was graded on a scale of zero to eight, with a score of 0 to 4 indicating "unfavorable attitude/perception " and a score of 5 to 8 indicating "favorable attitude/perception ". Practice towards VL was scored from zero to four. Finally, Practice against VL was measured on a scale of 0 to 4. Participants who received a score higher than 2 were deemed to have optimal/good VL practice. Participants with a score above 2 were considered to have optimal/good VL practice.

### Data analysis

Descriptive analysis was performed and the results were reported as frequencies and percentages. We conducted bivariable and multivariable regression analyses to determine the relationship between socio-demographic characteristics and participants’ KAP. The bivariable associations between independent variables and KAP indexes were investigated using binary logistic regression. All variables with a p-value <0.2 from the bivariable analysis were entered into the multivariable logistic regression model. Multivariable backward logistic regression analysis was used to determine the strongest predictor variable among participant’s characteristics and the KAP domains. Possible associations were measured using an adjusted odds ratio (AOR) with 95% CI and the p-value of less than 0.05 was considered statistically significant. Data analysis was performed using the Statistical Package for the Social Sciences (SPSS) version 27.0.

## Results

### Socio-demographic variables of the respondents

A total of 120 individuals participated. The mean(SD) age of participants was 37.03(9.8) years. The majority (68.3%) of the respondents were males, married (58.3%), and live in rural areas (80%). The educational characteristic shows that 40% were illiterates. Around 34% of the participants were working as laborers. Majority (59%) of the study participants were below poverty line (BPL card holders). Proportion of participants self-reported about their family members’ previous diagnosis with VL was 29% (Table 1).

**Table 1:** Socio- demographic variables of participants (N = 120).

Characteristics	Frequency	Percentage (%)
**Age**		
≤40	84	70
>41	36	30
**Gender**		
Male	82	68.3
Female	38	31.7
**Marital Status**		
Unmarried/single	30	25
Married	70	58.3
Divorced	06	05
Widowed	14	11.7
**Religion**		
Hindu	88	73.3
Muslims	24	20
Others	8	6.7
**Education**		
Illiterate	48	40
Primary school	29	24.2
Secondary school	24	20
Graduate/more	19	15.8
**Occupation**		
Agriculture worker	25	20.8
Business	7	5.8
Daily Labour	41	34.2
Govt./private job	17	14.2
Others	30	25
**Residence**		
Urban	24	20
Rural	96	80
**Family history of VL**		
Yes	35	29.2
No	85	70.8
**Below poverty line card**		
Yes	71	59.2
No	49	40.8
**HIV Duration**		
>3 years	46	38.3
≤3 years	74	61.7

### Knowledge about VL

Table 2 explains the participant’s knowledge about VL, where the majority 112(93.3%) of the study participants previously heard about VL and others 08(6.7%) excluded for the further evaluation. According to 69.6% of respondents, VL caused by sandfly and mosquito bites. A large proportion of respondents (85%) reported they couldn’t recognize a sandfly, and 42% said fever is the key symptom of VL. A substantial number of people (80.4%) were unaware of the sandfly breeding grounds. Awareness that VL is a preventable disease was found in 41.1% of participants, while 38.4% were unaware. Overall 72.3% had poor awareness of VL and its vector.

**Table 2:** Knowledge of study participants about visceral leishmaniasis (N = 112).

Variables	Categories	Frequency	Percentage (%)
Heard of VL (N = 120)	Yes	112	93.3
	No	8	6.7
Vector for VL (N = 112)	Sandfly *	36	32.1
	Housefly	3	2.7
	Mosquito	42	37.5
	Don’t know	31	27.7
Symptoms of VL ^Ұ^ (N = 112)	Splenomegaly*	11	9.8
	Stomach ache	4	3.6
	Skin pigmentation*	20	17.9
	Fever *	47	42
	Don’t know	30	26.8
Can you identify sandfly? (N = 112)	Yes*	27	24.1
	No	85	75.9
Knowing the Breeding habitats of sandfly (N = 112)	Yes*	22	19.6
	No	90	80.4
Biting time of vector (N = 112)	Dusk & dawn	15	13.4
	Midnight*	32	28.6
	Day time	6	8.9
	At any time	10	5.4
	Don’t know	49	43.8
Season for VL spread (N = 112)	Rainy season*	24	21.9
	Winter*	13	11.6
	Summer	33	29.5
	Don’t know	42	37.5
Is VL preventable? (N = 112)	Yes*	46	41.1
	No	23	20.5
	Don’t know	43	38.4
Overall Knowledge	Good	31	27.7
	Poor	81	72.3

*Correct responses were assigned a score 1 and other responses were scored 0.

^Ұ^ pointing out any symptom assigned a score of 1.

### Factors associated with knowledge

Bivariable analysis as indicated in Table 3 revealed that occupation and family history of VL was highly associated with the knowledge index. The multivariable regression analysis revealed that occupation and family history of VL were the two independent predictors of the knowledge index. Participants working in Govt /private offices (AOR: 7.821, 95% CI: 1.288–47.492) were more likely to have a better awareness of VL than other participants. Respondents with a family history of VL had a great understanding of the disease compared to their counterparts (AOR: 0.023, 95% CI: 0.005–0.097).

**Table 3:** Bivariable and multivariable logistic regression analysis showing predictors of knowledge levels.

Variables	Bivariable		Multivariable	
	Odds ratio (95% CI)	p-value	Adjusted odds ratio (95% CI)	p-value
**Age**				
≤40	1			
≥41	1.061 (0.410–2.743)	0.903		
**Gender**				
Male	1			
Female	1.097 (0.438–2.745)	0.843		
**Religion**				
Hindu	2.842 (0.326–24.792)	0.345		
Muslim	1.000 (0.087–11.525)	1.000		
Others	1			
**Residence**				
Urban	1			
Rural	1.026 (0.361–2.918)	0.962		
**Marital Status**				
Married	1.928 (0.493–7.485)	0.343		
Unmarried	2.000 (0.459–8.710)	0.356		
Divorced/Widowed	1			
**Education**				
Illiterate	1			
Primary school	1.973 (0.653–5.967)	0.229		
Secondary school	1.233 (0.387–3.928)	0.723		
Graduate/more	2.355 (0.725–7.642)	0.154		
**Occupation**				
Farmer	3.000 (0.968–9.302)	0.057	1.818 (0.357–9.255)	0.472
Govt/private job	4.286 (1.223–15.022)	**0.023** *	7.821 (1.288–47.492)	**0.025** *
Labour	0.079 (0.009–0.672)	**0.020** *	0.090 (0.008–1.023)	0.052
Others	1		1	
**VL Family history**				
Yes	1		1	
No	0.026 (0.008–0.083)	**<0.001** *	0.023 (0.005–0.097)	**<0.001** *
**Below poverty line**				
Yes	1.187 (0.502–2.806)	0.696		
No	1			
**HIV Duration**				
>3 years	1.913 (0.827–4.422)	0.129		
≤3 years	1			

CI = Confidence interval;

*p-value<0.05

### Attitude towards VL

Table 4 presents respondents’ attitudes regarding VL; The disease fatality was unknown to almost half of the study participants. Proportion of patients who believed that VL is a curable disease was 47.3%, whereas 42.9% of the patients believe that they do not have any risk to get the disease. Majority (72.3%) of the preferred public health care services as their first priority for VL treatment. A significant proportion of respondents (56.3%) believes that early diagnosis helps in the treatment of VL infection.

**Table 4:** Attitude towards VL and its treatability among study participants (N = 112).

Variables	Description	Frequency	Percentage (%)
VL is a curable disease	Yes*	53	47.3
	No	20	17.9
	I don’t know	39	34.8
VL will be fatal if not treated	Yes*	31	27.7
	No	25	22.3
	I don’t know	56	50
Community participation is crucial for VL control	Yes*	43	38.4
	No	19	17
	I don’t know	50	44
HIV infected people are more vulnerable to VL?	Yes *	29	25.9
	No	48	42.9
	I don’t know	35	31.3
Does living with a VL infected person raise the risk of getting a VL infection?	Yes*	33	29.5
	No	22	19.6
	I don’t know	57	50.9
Early diagnosis helps treatment of disease	Yes*	63	56.3
	No	13	11.6
	I don’t know	36	32.1
Inconsistent treatment affects recovery	Yes*	53	47.3
	No	16	14.3
	I don’t know	43	38.4
Preferred place of treatment	Public sector*	81	72.3
	Private sector	17	15.2
	Other	14	12.5
Overall attitude	Favorable	46	41.1
	Unfavorable	66	58.9

*Correct responses were assigned a score of 1 and other responses were assigned 0.

### Factors associated with attitude

In bivariable regression analysis as mentioned in Table 5, occupation was significantly associated with the attitude index of the participants (p-value<0.05). After removing insignificant predictor variables (p-value>0.2), all other variables were entered into the multivariable logistic regression model. The results revealed that age, and gender were the two independent predictor variables of attitude towards VL. Participants aged 41 years and above had significantly less favorable attitudes than their peers (AOR: 2.947, 95% CI: 1.250–8.267). Female participants (AOR: 2.733, 95% CI: 1.068–6.996) were more likely to have a favorable attitude than males.

**Table 5:** Bivariable and multivariable logistic regression analysis showing predictors of attitude levels.

Variables	Bivariable		Multivariable	
	Odds ratio (95% CI)	p-value	Adjusted odds ratio (95% CI)	p-value
**Age**				
≤40	2.600 (1.988–6.769)	0.050	2.947 (1.250–8.267)	**0.048** *
>41	1		1	
**Gender**				
Male	1		1	
Female	2.177 (0.99–5.047)	0.070	2.733 (1.068–6.996)	**0.036** *
**Religion**				
Hindu	5.721 (0.660–49.597)	0.113	4.531 (0.496–41.404)	0.181
Muslim	1.412 (0.131–15.266)	0.776	0.902 (0.076–10.687)	0.935
Others	1		1	
**Residence**				
Urban	1			
Rural	1.639 (0.609–4.407)	0.328		
**Marital Status**				
Married	2.786 (0.821–9.456)	0.100		
Unmarried	2.167 (0.569–8.255)	0.257		
Divorced/Widowed	1			
**Education**				
Illiterate	1			
Primary school	1.477 (0.539–4.045)	0.448		
Secondary school	0.967 (0.351–2.666)	0.948		
Graduate/more	1.289 (0.429–3.872)	0.651		
**Occupation**				
Farmer	2.600 (0.868–7.788)	0.088		
Govt/private job	4.033 (1.162–13.99)	**0.028** *		
Labour	0.978 (0.356–2.686)	0.965		
Others	1			
**VL Family history**				
Yes	1.832 (0.805–4.167)	0.149		
No	1			
**Below poverty line**				
Yes	1.109 (0.510–2.409)	0.794		
No	1			
**HIV Duration**				
>3 years	1		1	
≤3 years	2.024 (0.998–4.472)	0.081	2.352 (1.050–5.601)	0.053

CI = Confidence interval;

*p-value<0.05

### Participants’ practices toward VL prevention

Table 6 revealed that most (66%) of the respondents do not use bed nets, and some (37.5%) were sleeping outdoors. The widespread poor practice was observed among the participants. Around 28.6% of responders did not use any sandfly preventive strategies, and 35.7% had no idea how to care for VL patients.

**Table 6:** Practices toward the control and prevention of VL among the study participants (N = 112).

Variables	Categories	Frequency	Percentage (%)
Sleeping outdoor	Yes	42	37.5
	No*	70	62.5
Use of bed net	Yes *	38	33.9
	No	74	66.0
Prevention of sandfly^#^	Using bed nets	24	21.4
	Using mosquito repellents/coil	19	17.0
	Cleanliness	11	9.8
	Not use any prevention methods	32	28.6
	I don’t know	26	23.2
Patient care with VL	Use of bed net*	25	22.3
	Cleanliness	35	31.3
	Isolation of patients	12	10.7
	I don’t know	40	35.7
Overall Practice	Good	38	33.9
	Poor	74	66.1

*Correct response was assigned score 1 and other responses were scored 0.

^#^ Use of any preventive method has been assigned a score of 1.

### Factors associated with the practice

Bivariable analysis as indicated in Table 7 revealed that age, residence, occupation, family history of VL, and BPL status of the respondents were significantly associated with the overall practice, while in multivariable logistic regression analysis, respondents age, residence, occupation were found to be significantly associated with the practice. Participants working as labors (AOR: 0.248, 95% CI: 0.073–0.844) were less inclined to follow good preventive practices. There were increased odds of having good practice among participants aged 18–40 years (AOR: 6.866, 95% CI: 1.694–27.834) and those who reside in urban areas (AOR: 4.159, 95% CI: 1.317–13.139) than their peers.

**Table 7:** Bivariable and multivarible logistic regression analysis showing predictors of practice levels.

Variables	Bivariable		Multivariable	
	Odds ratio (95% CI)	p-value	Adjusted odds ratio (95% CI)	p-value
**Age**				
≤40	5.952 (1.665–21.274)	**0.006** *	6.566 (1.694–27.834)	**0.007** *
>41	1		1	
**Gender**				
Male	1.109 (0.460–2.678)	0.817		
Female	1			
**Religion**				
Hindu	4.080 (0.470–35.427)	0.202	4.816 (0.375–61.889)	0.228
Muslim	1.000 (0.087–11.525)	1.000	0.993 (0.062–15.953)	0.996
Others	1		1	
**Residence**				
Urban	3.954 (1.137–7.676)	**0.026** *	4.159 (1.317–13.139)	**0.015** *
Rural	1		1	
**Marital Status**				
Married	2.732 (0.712–10.48)	0.143		
Unmarried	2.702 (0.633–11.533)	0.180		
Divorced/Widowed	1			
**Education**				
Illiterate	1			
Primary school	0.753 (0.247–2.295)	0.618		
Secondary school	1.524 (0.551–4.214)	0.417		
Graduate/more	1.358 (0.439–4.197)	0.596		
**Occupation**				
Farmer	1.044 (0.356–3.060)	0.938	0.439 (0.125–1.547)	0.200
Govt/private job	1.299 (0.397–4.250)	0.665	1.273 (0.326–4.973)	0.729
Labour	0.320 (0.109–0.941)	**0.039** *	0.248 (0.073–0.844)	**0.026** *
Others	1		1	
**VL Family history**				
Yes	2.439 (1.051–5.657)	**0.038** *		
No	1			
**Below poverty line**				
Yes	2.739 (1.140–6.580)	**0.024** *		
No	1			
**HIV Duration**				
>3 years	1			
≤3 years	1.045 (0.470–2.324)	0.913		

CI = Confidence interval;

*p-value<0.05

## Discussion

Visceral leishmanias is endemic in Bihar for many decades, and its prevention and control are top priorities. In the era of VL elimination strategies, HIV-VL co-infection is one of the emerging challenges facing the elimination of VL. This study assessed the dynamics of knowledge, attitude and preventive practices of VL in HIV patients who receive care at the ART clinic. This is a preliminary study that assessed KAP about VL in HIV-positive people, thus filling a critical information gap.

A significant percentage of HIV patients (93.3%) had heard of VL, indicating that the disease is familiar in the community. This value is greater than our previous study conducted on PKDL patients (72.5%) [19] but lower than the population surveys conducted in Muzaffarpur dist., Bihar during 2006 [20] and 2010 [15] respectively. Only 32.1% of study participants knew that sand fly is the cause of VL transmission. Our finding is lower than the 68.1% found in a study in northwest Ethiopia [16], but greater than the 27.6% found in a cutaneous leshmaniasis(CL) study in Pakistan [21]. However, our findings are in agreement with those of Garapati et al. [19], who found that 32.5% of PKDL patients knew sandfly as the vector. Moreover, a considerable portion (80.4%) of the participants failed or unable to recognize the sandfly. This value exceeds the findings of a report on CL in South Ethiopia (69.32%) [17]. Awareness of clinical symptoms of VL is a prerequisite for early diagnosis and treatment in people infected with HIV. Most participants recognized fever as the key symptom of VL. These findings were comparable with those surveys conducted in Muzaffarpur district [15, 20].

Changing environmental and climatic circumstances could result in the expansion of geographic range of sandfly vector, thereby potentially increasing the danger of VL transmission [22]. Effective sandfly control requires the reduction of sandfly breeding habitats to reduce disease transmission. It is essential to educate people about the breeding habitat, biting time, and peak season of the disease in order to reduce human–vector contact [18, 20]. Furthermore, the present study discovered a lack of awareness about sandfly biting time, breeding habitat, and disease peak incidence time in people living with HIV in endemic areas. This is consistent with prior studies performed in non-HIV populations [18, 19, 21]. In this context, ENM (Ecological niche modeling) have shown to be a good tool for determining environmental predictors that favour parasite transmission and has been used to forecast epidemics, which is crucial for health systems to deal with such outbreaks [23, 24]. Although a study in Bangladesh found that ENM is particularly effective in disease prediction, the researchers also found that environmental variables such as precipitation during the warmest quarter of the year, land surface temperature (LST), and the normalised differential water index (NDWI) can all be used to predict VL in the study area [24]. The results of the ENM can also be used to predict probable sandfly dispersion and assist health professionals in adopting informed decisions and tailored interventions by creating awareness in areas where the disease is likely to resurface. ENM should therefore be regarded a useful tool in vector surveillance and sand fly control management. We believe that understanding the environmental risk factors associated with the disease is essential for combating this deadly disease.

Respondent’s knowledge about the disease preventability was much lower than the findings of studies in the non-HIV respondents from other countries [16, 17, 25]. When the respondents’ overall knowledge in this study was taken into account, 72.3% had a poor understanding of it. This value is higher than that of a study carried out in Madhepura district of Bihar, where 56.1% had poor knowledge. Our findings are in contrast with earlier cross-sectional studies in Ethiopia, which found that respondents have a clear understanding of VL [16, 17].

A large portion (72.3%) of study participants preferred public health centers as their first choice for treatment. This could be attributed to the fact that the public sector is more accessible and less expensive than the private sector, along with their experience with the Indian government’s free ART care. Our findings were in contrast with those previously reported from Muzaffarpur dist, Bihar [20]. About 47.3% of respondents believed that VL can be cured and had a positive attitude about it. However, our results were in contrast with Berhe et al., who found that 90.5% of respondents believe VL is treatable [17]. Community involvement was not well supported, with just 38.4% believing it is important for disease prevention. It is impossible to prevent VL from endemic areas without active community participation. Furthermore, since the majority of VL epidemics occur in rural areas, all inhabitants ought to be responsible for cleaning up their homes and surroundings and clearing sandfly breeding sites. In contrast to our findings, some studies are reported in which the general public believes that the community also has the responsibility in controlling VL [15, 16]. The Majority of the participants (50%) had a perception that they do not get VL. This indicates that the majority of responders are ignorant that VL is an opportunistic infection in HIV-positive individuals. People living with HIV in endemic areas should be aware that VL is a communicable disease that can be lethal if left untreated. According to our research, most HIV-positive people have a negative attitude toward VL (understanding early diagnosis helps the treatment of disease, understanding fatality of the disease if not treated). Out of 112 respondents, 46 (41.1%) had a favorable attitude about VL, which is in contrast with various studies conducted in a normal population [16, 17].

Most (66.1%) of the participants used to sleep without bed nets, and 37.5% had a habit of sleeping outdoors, putting them at high risk of contracting VL. This may be attributed to the respondents’ low socio-economic status. These findings are comparable to that of a study on CL in South Ethiopia, which found that 40.8% of people used to sleep outdoors [26]. Only 33.9% of the respondents in this study had strong preventive strategies, while the remaining respondents did not practice well for disease control and prevention. This percentage is lower than that of a study reported from Ethiopia [17] where 53.4% had good preventive practices.

The multivariable model found that occupation and VL family history were important determinants of good VL knowledge. However, only age and gender were reported as independent predictors of knowledge towards CL from Yemen [27]. Occupation of the participants showed a significant positive influence on the knowledge index. This finding was in line with the study conducted in Bihar [18]. Participants working in Govt. /private firms were more likely to have good knowledge towards VL. This could be because the government and private-sector employees are more likely to participate in the health-awareness campaigns at workplace. Respondents who had a family history of VL were more knowledgeable of the disease than their peers. This finding is concurrent with a study conducted in Ethiopia where households with a positive family history of VL had better knowledge [28]. This indicating that VL patients served as a primary root of information in the community.

Adults whose age was 18–40 range were at higher odds of having a good attitude and practice than older participants (>41 years). The possible reason for this might be due to the fact that participants in the age range of 18–40 years are in the adulthood stage, which allows them to be socially proactive and practice well. The finding also indicates that female participants had a better attitude towards VL as compared to their male counterparts. This is in agreement with the study conducted in northwest Ethiopia [29]. VL considered a poverty-related disease, primarily affecting people living in rural areas [18]. On the other hand, participants in our study who resided in urban regions had good practice than those who lived in rural areas. Overall, the current study found out that HIV patients had limited knowledge (72.3%), attitude (58.9%), and practice (66.1%) towards VL. In comparison with other similar studies, our study subjects had lower KAP scores than non-HIV infected populations [16–18]. This could be because, PLHIV may consider HIV to be more stigmatizing than VL, hence VL is less important to them. Furthermore, HIV patients have a plethora of psychological issues that may function as a roadblock to adequate KAP for VL. Overall, the findings emphasize the importance of educational campaigns and persistent activities towards behavioral change in HIV patients living in endemic areas.

There are few drawbacks to this research. Firstly, a matched HIV-negative control group was unavailable for comparison. This was a single-center hospital study; its generalizability may be compromised. Since the patients who participated in the study were drawn from a convenience sample from the ART clinic, there may have been a selection bias. This study was a cross-sectional survey; therefore only association can be determined and not causations. We used bivariable logistic regression to analyze data, which required the respondents’ KAP scores to be dichotomized, resulting in the inclusion or exclusion of some respondents with scores close to the median. We suspect that several factors with significant differences in scores may have been missed as a result of this grouping. The findings of the study cannot be inferred to other populations in the country due to intercultural and socio-demographic disparities. However, we were unable to control potential confounders by randomization, restriction, and matching of the dependent variable, thus we used a multivariable logistic regression model to control the effect of confounders. Finally, the confidence intervals for several variables are too wide, indicating the inadequacy of sample size. Increasing the sample size, on the other hand, may assist overcome this limitation.

## Conclusion

This study assessed the dynamics of knowledge, attitude and preventive practices of VL in HIV patients who receive care at an ART clinic by using a pretested questionnaire. This is a preliminary study that evaluated KAP about VL in HIV-positive people, addressing a significant information gap. In general, the finding of the study showed that the PLHIV had insufficient knowledge about disease transmission, vector breeding site, time or season of bite and prevention strategies of VL. In order to limit human–vector interaction, frontline health workers in endemic areas should provide accurate information to PLHIV about VL transmission, environmental risk factors, and necessary protective measures. Besides that, more than half of those respondents had a negative attitude toward VL and poor preventive practices. Occupation and VL family history were the strongest predictors of good knowledge. On the other hand participants working as labors, those over the age of 40, and those living in rural areas were less likely to practice good preventive measures. Treatment of VL in HIV patients remains fraught with difficulties. Those who recognize the clinical symptoms may seek medical attention earlier than others. Health education and counseling programs in ART centers can be helpful in raising awareness about VL. HIV-VL co-infection in endemic areas can be avoided with proper awareness and a preventive attitude toward VL in PLHIV.

## Supporting information

S1 File KAP English questionnaire. (DOCX)

S2 File KAP Hindi questionnaire. (DOCX)
