# Supplementary material for: Knowledge, attitude and practices towards visceral leishmaniasis among HIV patients: A cross-sectional study from Bihar, India
Source: PLoS One. 2021 Aug 17;16(8):e0256239. doi: 10.1371/journal.pone.0256239 (PMC8370793; doi:10.1371/journal.pone.0256239)
Supplement: S1 File — (DOCX) [file pone.0256239.s001.docx]

**Date:**

**Sociodemographic details**

**1. Age:**

**2. Sex:**

**3. Education:**

**4. Occupation:**

**5. Marital status:**

**6. Religion:**

**7. Residence:**

**8. BPL status:**

**9. Family history VL:**

**10. Duration of HIV:**

**Knowledge on viceral leishmaniasis disease**

1. Have you ever heard of visceral leishmaniasis disease?

Yes

No

I don’t know

1. Do you know the vector of visceral leishmaniasis disease?

Sand fly

House fly

Mosquito

I don’t know

1. Do you know the symptoms of visceral leishmaniasis?

Splenomegaly

Fever

Stomache ache

Skin pigmentation

I don’t know

1. Can you identify a sandfly?

Yes

No

1. Do you know biting time of sandfly?

During midnight

During day time

At any time

I don’t know

1. Do you know the season of Visceral leishmaniasis spread?

Summer

Winter

Raining season

I don’t know

1. Do you know breeding places of sand fly?

Yes

No

I don’t know

1. Is visceral leishmaniasis preventable?

Yes

No

I don’t know

**Attitude towards viceral leishmaniasis**

1. Is visceral leishmaniasis a curable disease?

Yes

No

I don’t know

1. Do you consider yourself at risk of visceral leishmaniasis?

Yes

No

I don’t know

1. Does living with a visceral leishmaniasis infected person raise the risk of getting an infection?

Yes

No

I don’t know

1. Is visceral leishmaniasis fatal, if left it untreated?

Yes

No

I don’t know

1. Is it possible to control visceral leishmaniasis through community participation?

Yes

No

I don’t know

1. Can an early diagnosis aid in the treatment of visceral leishmaniasis ?

Yes

No

I don’t know

1. Is inconsistent treatment has an impact on visceral leishmaniasis recovery?

Yes

No

I don’t know

1. What is your first choice of health-care system for the treatment of suspected visceral leishmaniasis?

Public sector

Private sector

Others

**Practices toward the prevention of visceral leishmaniasis**

1. Do you use bed nets while sleeping?

Yes

No

1. Do you sleep in outdoors?

Yes

No

1. What preventive measures do you take to avoid sandfly bites?

By using bed nets

By using Insecticides

By maintaining Cleanliness

I don’t use any prevention methods

I don’t know

1. How do you take care of visceral leishmaniasis patient?

Use bed nets

Cleanliness

Isolation of patient

I don’t know
