# Supplementary material for: Knowledge, attitude and practices towards visceral leishmaniasis among HIV patients: A cross-sectional study from Bihar, India
Source: PLoS One. 2021 Aug 17;16(8):e0256239. doi: 10.1371/journal.pone.0256239 (PMC8370793; doi:10.1371/journal.pone.0256239)
Supplement: S2 File — (DOCX) [file pone.0256239.s002.docx]

तारीख:

सामाजिक-जनसांख्यिकीय विवरण

1. उम्र:

2. सेक्स:

3. शिक्षा:

4. परोजगार:

5. वैवाहिक स्थिति:

6. धर्म:

7. निवास:

8. बीपीएल स्थिति:

9. पारिवारिक इतिहास वीएल:

10. एचआईवी की अवधि:

कालाजार रोग पर ज्ञान

1.क्या आपने कभी कालाजार रोग के बारे में सुना है?

हाँ

नहीं

2. क्या आप कालाजार रोग के वाहक को जानते हैं?

रेत मक्खी/ सैंड फ्लाई/ बालू मक्खि

हाउस फ्लाई

मच्छर

मुझें नहीं पता

3. क्या आप कालाजार के लक्षण जानते हैं?

तिल्ली/प्लीहा का बढ़ना

बुखार

पेट का दर्द

त्वचा रंजकता

मुझें नहीं पता

4. क्या आप बालू मक्खि की पहचान कर सकते हैं ?

हाँ

नहीं

5. क्या आप बालू मक्खि/ रेत मक्खी के काटने का समय जानते हैं ?

आधी रात के दौरान

दिन के समय

किसी भी समय

मुझें नहीं पता

6. क्या आप जानते हैं कालाजार किस मौसम में फैलता है?

गर्मी

सर्दी

वर्षा ऋतु

मुझें नहीं पता

7. क्या आप बालू मक्खियों के प्रजनन स्थलों के बारे में जानते हैं?

हाँ

नहीं

मुझें नहीं पता

8. क्या कालाजार को रोका जा सकता है?

हाँ

नहीं

मुझें नहीं पता

**कालाजार के नियंत्रण के लिए** **प्रतिभागियों के बीच कालाजार के प्रति मनोभाव**

1. क्या कालाजार एक इलाज योग्य बीमारी है?/ क्या कालाजार पूरी तरह से ठीक होने वाली बीमारी है?

हाँ

नहीं

मुझें नहीं पता

2. क्या आप खुद को कालाजार के जोखिम में मानते हैं?

हाँ

नहीं

मुझें नहीं पता

3. क्या कालाजार संक्रमित व्यक्ति के साथ रहने से कालाजार होने का खतरा बढ़ जाता है?

हाँ

नहीं

मुझें नहीं पता

4. क्या कालाजार एक जानलेवा बीमारी है, अगर इलाज न किया जाए?

हाँ

नहीं

मुझें नहीं पता

5. क्या सामुदायिक भागीदारी से कालाजार पर नियंत्रण संभव है?

हाँ

नहीं

मुझें नहीं पता

6. क्या कालाजार के इलाज में शीघ्र निदान सहायता कर सकता है?

हाँ

नहीं

मुझें नहीं पता

7 क्या इलाज में असंगति का कालाजार के ठीक होने पर असर पड़ता है?

हाँ

नहीं

मुझें नहीं पता

8. संदिग्ध कालाजार के इलाज के लिए स्वास्थ्यचर्या प्रणाली की आपकी पहली पसंद क्या होगी?

सार्वजनिक क्षेत्र

निजी क्षेत्र

अन्य

**कालाजार के नियंत्रण और रोकथाम की दिशा में अभ्यास**

1. क्या आप सोते समय बेड नेट का इस्तेमाल करते हैं?

हाँ

नहीं

2. क्या आप बाहर सोते हैं?

हाँ

नहीं

3. क्या आप बालू मक्खि के काटने से बचने के लिए कोई निवारक उपाय अपनाते हैं?

बिस्तर जाल का उपयोग करके

कीटनाशकों के प्रयोग से

स्वच्छता बनाए रखने से

मैं किसी भी रोकथाम के तरीकों का उपयोग नहीं करता

मुझें नहीं पता

4. आप कालाजार के मरीज की देखभाल कैसे करते हैं?

मच्छरदानी का प्रयोग करके

स्वच्छता बनाए रखने से

रोगियों को अलग करके

मुझें नहीं पता
